# Laparoscopic gastrectomy versus open gastrectomy for elderly patients with gastric cancer: a systematic review and meta-analysis

**DOI:** 10.1186/s12957-016-0859-8

**Published:** 2016-03-31

**Authors:** Jin-fa Wang, Song-ze Zhang, Neng-yun Zhang, Zong-yang Wu, Ji-ye Feng, Li-ping Ying, Jing-jing Zhang

**Affiliations:** Department of General Surgery, Yinzhou People’s Hospital, Yinzhou Hospital Affiliated to Medical School of Ningbo University, 251 Baizhang Road, Ningbo, 315040 Zhejiang People’s Republic of China

**Keywords:** Gastric cancer, Laparoscopy, Gastrectomy, Elderly, Meta-analysis

## Abstract

**Background:**

The objective of this study was to evaluate the feasibility, safety, and potential benefits of laparoscopic gastrectomy (LG) comparing with open gastrectomy (OG) in elderly population.

**Methods:**

Studies comparing LG with OG for elderly population with gastric cancer, published between January 1994 and July 2015, were identified in the PubMed, Embase, and ISI Web of Science databases. Operative outcomes (intraoperative blood loss, operative time, and the number of lymph nodes harvested) and postoperative outcomes (time to first ambulation, time to first flatus, time to first oral intake, postoperative hospital stay, postoperative morbidity) were included and analyzed. The Newcastle-Ottawa Scale was used to assess the quality of the pooled study. A funnel plot was used to evaluate the publication bias.

**Results:**

Seven studies totaling 845 patients were included in the meta-analysis. LG in comparison to OG showed less intraoperative blood loss (weighted mean difference (WMD) −127.47; 95 % confidence interval (CI) −202.79 to −52.16; *P* < 0.01), earlier time to first ambulation (WMD −2.07; 95 % CI −2.84 to −1.30; *P* < 0.01), first flatus (WMD −1.04; 95 % CI −1.45 to −0.63; *P* < 0.01), and oral intake (WMD −0.94; 95 % CI −1.11 to −0.77; *P* < 0.01), postoperative hospital stay (WMD −5.26; 95 % CI −7.58 to −2.93; *P* < 0.01), lower overall postoperative complication rate (odd ratio (OR) 0.39; 95 % CI 0.28 to 0.55; *P* < 0.01), less surgical complications (OR 0.47; 95 % CI 0.32 to 0.69; *P* < 0.01), medical complication (OR 0.35; 95 % CI 0.22 to 0.56; *P* < 0.01), incisional complication (OR 0.40; 95 % CI 0.19 to 0.85; *P* = 0.02), and pulmonary infection (OR 0.49; 95 % CI 0.26 to 0.93; *P* = 0.03). No significant differences were observed between LG and OG for the number of harvested lymph nodes. However, LG had longer operative times (WMD 15.73; 95 % CI 6.23 to 25.23; *P* < 0.01).

**Conclusions:**

LG is a feasible and safe approach for elderly patients with gastric cancer. Compared with OG, LG has less blood loss, faster postoperative recovery, and reduced postoperative morbidity.

## Background

Gastric cancer remains one of the leading causes of cancer-related death worldwide, especially in East Asia [1–4]. Radical gastrectomy is the mainstay of the curative treatment for gastric cancer. As life expectancy has increased consistently, inevitably, an increasing number of aged people with gastric cancer are anticipated to undergo gastrectomy with the goal of radical treatment [5]. Characteristics of elderly patients such as declining physiological function and poor nutritional status, together with severe surgical traumas of radical gastrectomy, appear to result in higher postoperative morbidity, prolonged hospital stay, increasing financial burden, and even higher postoperative mortality. Approaches with less surgical traumas and milder acute inflammation response are urged.

Despite of controversy, laparoscopic gastrectomy (LG) has been developed as an innovation in the management of gastric cancer [6–8]. Many previous studies including several randomized clinical trials on LG have referred to its surgical benefits of less invasiveness [9–12]. In addition, recent advances in laparoscopic instruments and accumulating surgical experience impelled surgeons to apply LG in locally advanced gastric cancer. Growing evidences have suggested LG was able to achieve equivalent oncological outcomes as open gastrectomy (OG) in both early and advanced gastric cancer [13, 14]. Though concerning the pneumoperitoneum, LG has been gradually performed in elderly population. Researches specifically studying the application of LG in elderly population are limited. Hence, we comprehensively collected relevant evidences and conducted this systematic review with meta-analysis to assess the feasibility, safety, and potential benefits of LG in elderly population.

## Methods

### Search strategy

Articles published from January 1994 to July 2015 were searched in the PubMed, Embase, and ISI Web of Science databases. The search strategy was performed using the following terms: “gastric cancer,” “gastric adenocarcinoma,” “gastric neoplasms,” “laparoscopy,” “laparoscopic,” “elderly,” “old,” and “aged.” All abstracts retrieved from the electronic databases were screened. Then, the full texts were retrieved when abstracts were relevant. The references of all relevant articles were also manually searched for potentially relevant studies.

### Study selection

Eligibility criteria included the following: (1) histologically confirmed gastric cancer; (2) published studies comparing LG with OG for gastric cancer; (3) inclusion of elderly patients; and (4) availability of data on information of at least three outcome measures. Exclusion criteria included the following: (1) recurrent gastric cancer; (2) hand-assisted surgery or robotic surgery; (3) combined with other malignancies; (4) abstracts presented at meetings, review articles, case report, or letters; and (5) palliative gastrectomy. If more than one study of a single institution existed, the study with the most recent or the most informative data was included unless the relevant outcomes were only published in earlier version.

### Data extraction and quality assessment

Two reviewers independently extracted and checked data using a standard form. Disagreements in data extraction were resolved through discussion and consensus of the study team. The following data were extracted from each study: study name, study period, sample size, age, body mass index (BMI), comorbidity, extent of lymph node dissection, method of gastrectomy, tumor size, tumor location, operation time, intraoperative blood loss, number of harvested lymph nodes, time to first flatus, time to first oral intake, length of postoperative hospital stay, and postoperative complications. The qualities of studies were evaluated using the Newcastle-Ottawa Quality Assessment Scale (NOS) [15]. Studies with a score equal to or higher than six stars were considered methodologically sound.

### Statistical methods

Dichotomous variables were evaluated by using odds ratio (OR) with a 95 % confidence interval (95 % CI), and continuous variables were analyzed using the weighted mean difference (WMD) with a 95 % CI. If the study provided medians and ranges instead of means and standard deviations (SDs), the means and SDs were calculated using the method described by Hozo et al. [16]. Heterogeneity was evaluated by Cochran’s Q-statistic and *I*^2^ [17]. If data was not significantly heterogeneous (*P* > 0.05 or *I*^2^ < 50 %), the pooled effects were calculated using a fixed model [18]. Otherwise, the pooled effects were calculated using a random-effects model [19]. Publication bias was evaluated visually using a funnel plot. All data were analyzed using the Review Manager Version 5.0 (The Cochrane Collaboration, Oxford). *P* < 0.05 was considered statistically significant.

## Results

### Study characteristics

**Fig. 1:**
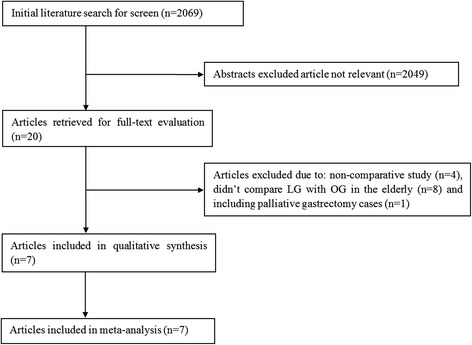
Flow chart of the identification and inclusion of studies

The search strategy initially identified 2069 studies. After exclusion of irrelevant studies, 20 potentially relevant articles were obtained for assessment. Thirteen studies were excluded due to non-comparative studies, did not compare LG with OG, and including palliative gastrectomy cases. Finally, seven studies (three from Japan and four from China) published between 2004 and 2015 were included [20–26]. The PRISMA flowchart of literature review is shown in Fig. 1. The characteristics of these seven studies are summarized in Table 1. A total of 845 patients from East Asia were pooled in this meta-analysis: 422 in the LG group and 423 in the OG group. Patients more than 70 years old were categorized as elderly patients in four studies [20, 21, 24, 25], more than 65 years old in two studies [22, 23], and more than 75 years old in one study [26]. Patients from Japan mostly suffered early gastric cancer and underwent D1 or D1+ lymphadenectomy, while the majority of patients from China suffered advanced gastric cancer and underwent D2 lymphadenectomy. Three studies compared the prognostic outcomes and demonstrated no significant difference between LG and OG. Oncological outcomes of included studies are showed in Table 2. All seven studies were methodologically sound with no less than six stars (Table 3).

**Table 1:** Characteristics of included studies

Study	Period	Country	Sample size	Gender		Age		BMI		Comorbidity		Age cutoff point	Comparability of baseline characteristics
				LG	OG	LG	OG	LG	OG	LG	OG		
Yasuda	1994–2003	Japan	73	26/19	17/11	75.7	77.2	NR	NR	55.60 %	67.90 %	70	abdfi
Mochiki	1998–2004	Japan	46	20/10	14/2	75.2	74.3	NR	NR	43.00 %	25.00 %	70	abdei
Meng	2007–2009	China	225	88/28	107/32	71.4	71.5	21.9	22.3	49.10 %	54 %	65	abcdefghi
Hu	2007–2012	China	233	74/35	83/41	72.4	71.8	NR	NR	51.40 %	54.80 %	65	abdefgh
Li	2008–2009	China	108	36/18	30/24	78.6	76.5	NR	NR	85.20 %	81.50 %	70	abdg
Qiu	2012–2013	China	64	25/5	22/12	74.4	75.6	21.6	21.6	NR	NR	70	abcdefgh
Suzuki	2000–2011	Japan	66	28/10	18/10	78.5	77	22.5	23	73.70 %	85.70 %	75	abcdgi

*BMI* body mass index, *LG* laparoscopic gastrectomy, *OG* open gastrectomy, *NR* not reported, *a* gender, *b* age, *c* BMI, *d* comorbidity, *e* tumor size, *f* tumor location, *g* tumor stage, *h* type of gastrectomy, *i* type of anastomosis

**Table 2:** Oncological outcomes of included studies

Study	Country	Group	Tumor stage	Extent of LND	Number of retrieved lymph nodes	Length of follow-up	Prognostic outcomes
			EGC/AGC	D1/D1+/D2			
Yasuda	Japan	LG	44/1	45/0/0	NR	NR	NR
		OG	24/4	28/0/0	NR	NR	NR
Mochiki	Japan	LG	29/1	0/30/0	NR	Median 40 m	5-year OS rate 95.7 %, 5-year DFS rate 96 %
		OG	14/2	0/16/0	NR	NR	NR
Meng	China	LG	38/78	0/0/116	29 ± 11	2~48 m	Median survival time 23 m
		OG	41/98	0/0/139	27 ± 10		Median survival time 22.5 m
Hu	China	LG	24/85	0/0/109	31.4 ± 14.2	2~56 m	1-year OS rate 91.0 %, 3-year OS rate 73.7 %, 5-year OS rate 54.5 %
		OG	25/99	0/0/124	32.6 ± 11.7		1-year OS rate 92.9 %, 3-year OS rate 77 %, 5-year OS rate 59.2 %
Li	China	LG	3/51	0/0/54	27.8 ± 3.9	36 m	1-year OS rate 85.2 %, 3-year OS rate 55.6 %
		OG	5/49	0/0/54	26.7 ± 4.6		1-year OS rate 81.5 %, 3-year OS rate 57.4 %
Qiu	China	LG	0/30	0/0/30	30.2 ± 12.0	NR	NR
		OG	0/34	0/0/34	28.1 ± 11.8	NR	NR
Suzuki	Japan	LG	37/1	6/30/2	NR	Median 42 m	Three died from pneumonia, one was lung cancer-related,and one was while bathing
		OG	26/2	12/12/4	NR		One patient died from gastric cancer and three died from cerebrovascular disease

*EGC* early gastric cancer, *AGC* advanced gastric cancer, *LND* lymph node dissection, *LG* laparoscopic gastrectomy, *OG* open gastrectomy, *NR* not reported, *OS* overall survival, *DFS* disease-free survival

**Table 3:** Quality assessment of included studies

Study	Selection				Comparability	Outcomes			Total
	Representativeness of exposed cohort	Selection of nonexposed cohort	Ascertainment of exposure	Outcome not present at the start of the study		Assessment of outcomes	Length of follow-up	Adequacy of follow-up	
Yasuda	*	*	*	*	**	*			*******
Mochiki	*	*	*	*	**	*	*	*	*********
Meng	*	*	*	*	**	*	*		********
Hu	*	*	*	*	**	*	*		********
Li	*	*	*	*	*	*	*		*******
Qiu	*	*	*	*	**	*			*******
Suzuki	*	*	*	*	**	*	*		********

*It stands for one score in the assessment of study quality

### Operative outcomes

**Fig. 2:**
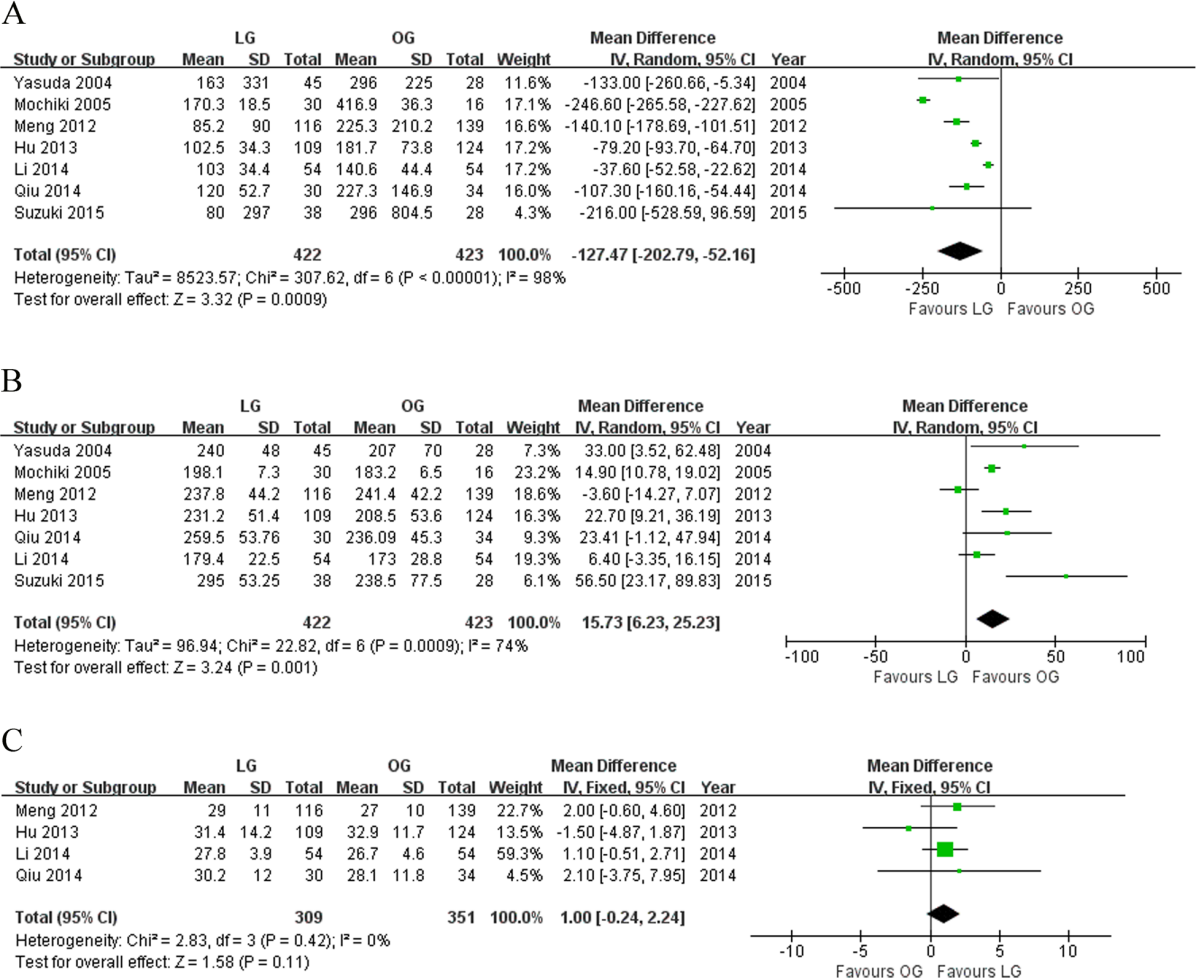
Meta-analyses of operative outcomes. **a** Intraoperative blood loss. **b** Operative time. **c** Number of harvested lymph node

All seven pooled studies reported the operation time and intraoperative blood loss. Our meta-analysis suggested LG was associated with a reduction in intraoperative blood loss (WMD −127.47; 95 % CI −202.79 to −52.16; *P* < 0.01; Fig. 2a), although longer operation time was also observed (WMD 15.73; 95 % CI 6.23 to 25.23; *P* < 0.01; Fig. 2b). In addition, LG achieved equivalent lymph nodes compared with OG (WMD 1.00; 95 % CI −0.24 to 2.24; *P* = 0.11; Fig. 2c).

### Postoperative outcomes

**Fig. 3:**
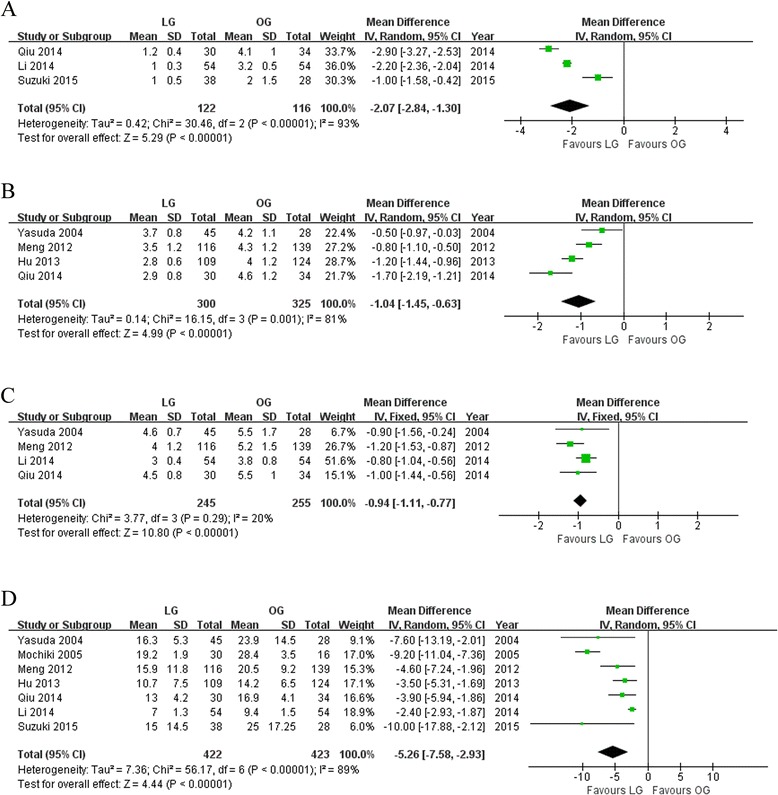
Meta-analyses of postoperative recovery **a** Time to first ambulation. **b** Time to first flatus. **c** Time to first oral intake. **d** Postoperative hospital stay

Patients in the LG group have earlier time to ambulation than those in the OG group by about 2 days (WMD −2.07; 95 % CI −2.84 to −1.30; *P* < 0.01; Fig. 3a). The LG group also had favored time to first flatus (WMD −1.04; 95 % CI −1.45 to −0.63; *P* < 0.01; Fig. 3b), time to resume oral intake (WMD −0.94; 95 % CI −1.11 to −0.77; *P* < 0.01; Fig. 3c), and postoperative hospital length (WMD −5.26; 95 % CI −7.58 to −2.93; *P* < 0.01; Fig. 3d).

**Fig. 4:**
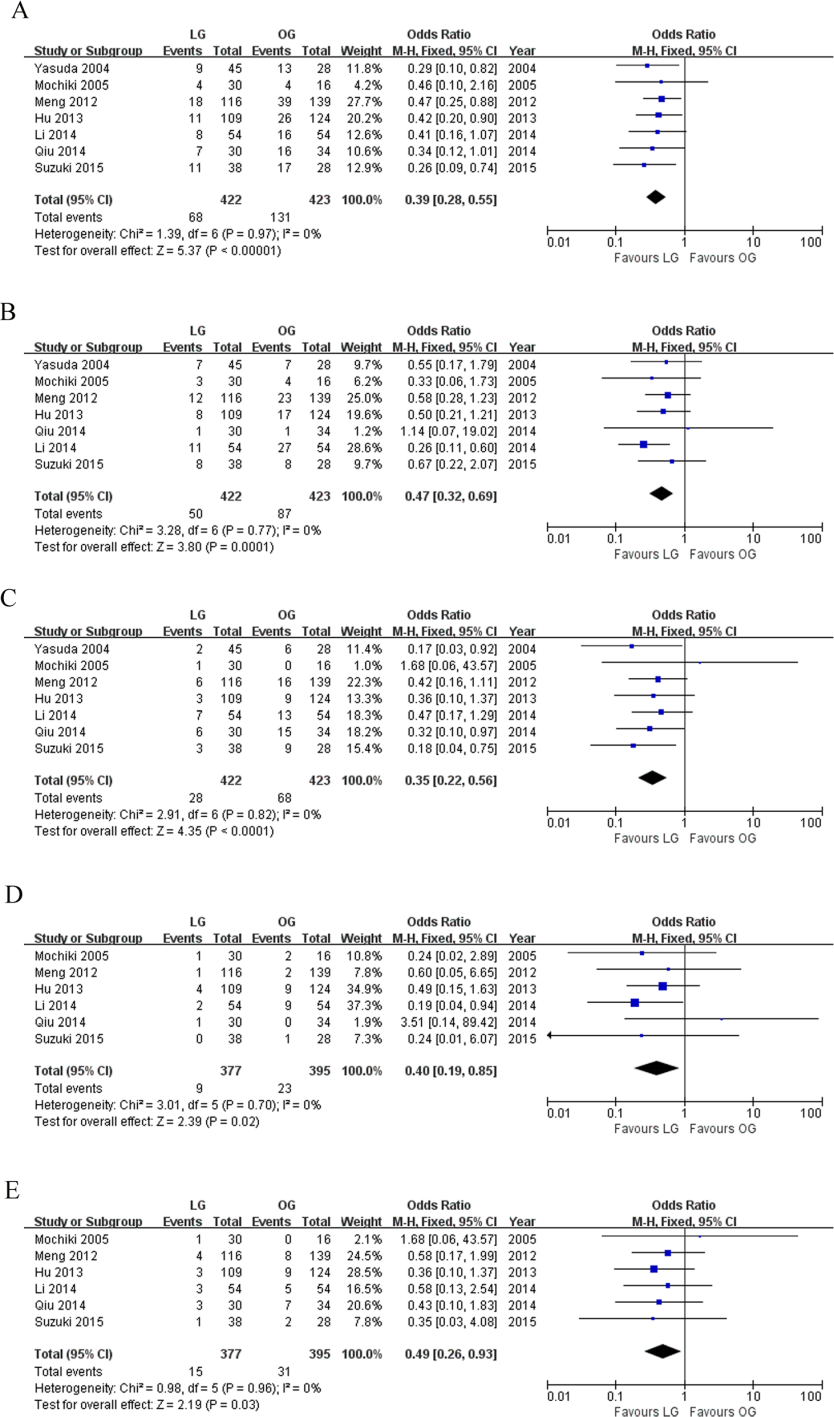
Meta-analyses of postoperative complication. **a** Overall postoperative complication. **b** Surgical complication. **c** Medical complication. **d** Incisional complication. **e** Pulmonary infection

The postoperative complications were recorded in all studies. The LG group had lower overall postoperative complication rate than the OG group (OR 0.39; 95 % CI 0.28 to 0.55; *P* < 0.01; Fig. 4a). In detail, LG comparing with OG showed reduced surgical complications (OR 0.47; 95 % CI 0.32 to 0.69; *P* < 0.01; Fig. 4b) and medical complication (OR 0.35; 95 % CI 0.22 to 0.56; *P* < 0.01; Fig. 4c). Further analysis also revealed that the LG group was associated with lower incisional complication (OR 0.40; 95 % CI 0.19 to 0.85; *P* = 0.02; Fig. 4d) and pulmonary infection rate (OR 0.49; 95 % CI 0.26 to 0.93; *P* = 0.03; Fig. 4e).

### Sensitivity analysis and publication bias

**Fig. 5:**
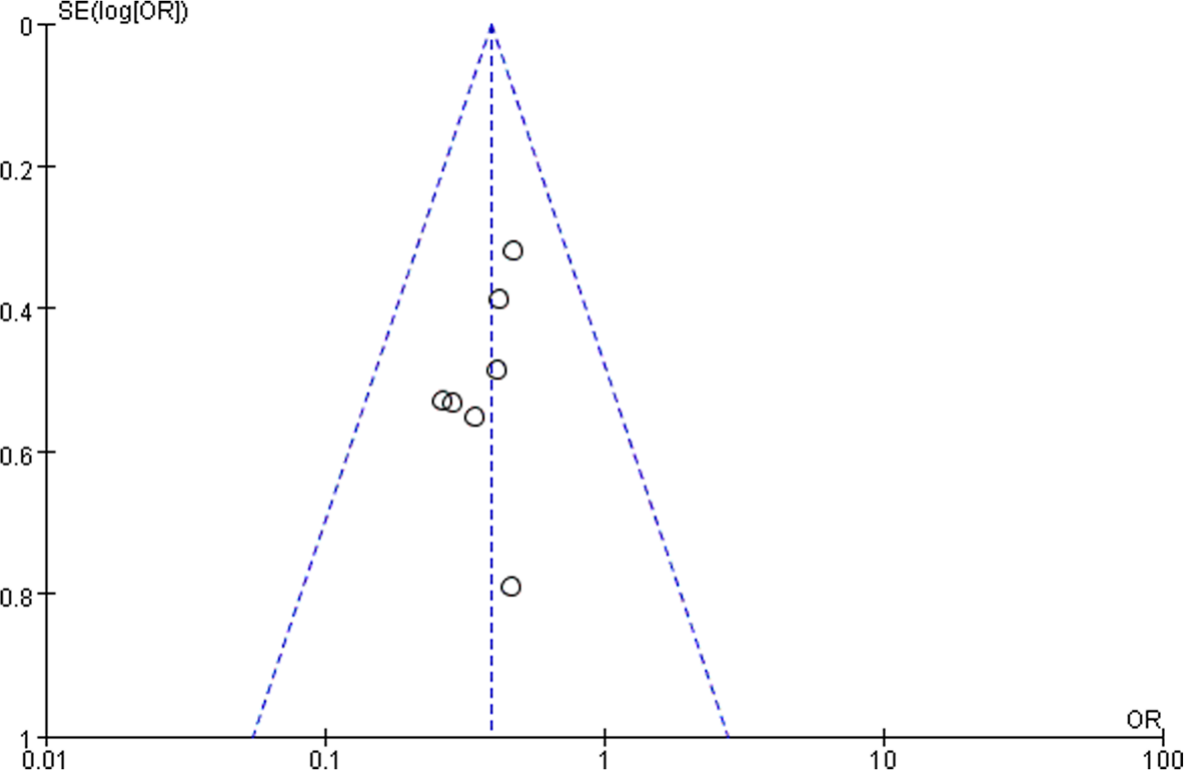
Funnel plot of meta-analysis

Sensitivity analyses were conducted by exclusion of the highest weighted study in each pooled analysis. These exclusions did not alter the results obtained in cumulative analyses. Funnel plot based on the overall postoperative complication was performed to assess publication bias. No significant publication bias was detected by visual inspection of the funnel plot in which the pooled studies were almost symmetrical and none of them was outside the 95 % CI (Fig. 5).

## Discussion

With continuing growth of the elderly population, more elderly patients undergo gastrectomy for gastric cancer. Generally, the elderly patients are usually accompanied with impaired physiological function, clinically presenting as a higher incidence of comorbidities, which is likely to have adverse effects on perioperative outcomes and result in postoperative complications or even death [27, 28]. Lee et al. reported postoperative morbidity including systemic complication, and severe complication showed higher tendency with increased age [29]. Minimally invasive and enhanced recovering approaches are urged for this special population. Surgeons have applied laparoscopic technique in nearly all abdominal surgeries, including gastric cancer. However, convincing evidences remain lacking which impels us to conduct this meta-analysis. We found that patients who underwent LG were associated with less blood loss, faster recovery, and less postoperative morbidity as compared with its open counterpart.

Postoperative complications of gastrectomy result in several events, including longer hospital stays, increased medical expenses, delayed adjuvant chemotherapy, and oncological outcomes. Kubota et al. revealed that postoperative complications that can cause prolonged inflammation result in shorter overall survival (OS) and worse disease-specific mortality even if the tumor is resected curatively [30]. One of the main concerns with LG in elderly population is the possibility of cardiopulmonary complication related to pneumoperitoneum. Whereas our meta-analysis found that patients who underwent LG have lower risk of medical complication, especially the postoperative pneumonia, which was in conformity with several reports [31]. Milder pain associated with LG encourages patients to expectorate and to start postoperative activity earlier. Suzuki et al. also reported that the cardiopulmonary adverse effects due to pneumoperitoneum were transitory and normalized during the intraoperative period and were acceptable even among decrepit elderly patients having cardiopulmonary disease [26]. Avoidance of the large incision and completing the gastrointestinal reconstruction with or without a mini-laparotomy reduces the risk of wound infection. Smaller incision and meticulous manipulation helped to remit the postoperative pain and reduce surgical stress. Okholm et al. reported that LG attenuates the postoperative immune response compared to open surgery [32]. From this point, patients who underwent LG were able to have enhanced bowel recovery. Our study also found that the LG group had shorter bedbound time, time to first flatus, time to resume oral intake, and length of hospital stay.

In accordance with previous studies [33–35], the LG group had a longer operation time by about 15 min in our study. Longer operation time was considered as an adverse factor of surgical outcomes. Miki et al. reported that patients with extended operation time had higher risk of severe postoperative complication [36]. Owing to the introduction of automatic-sewn techniques and avoidance of open and closure of conventional surgical incision as OG, operation time of LG gradually reduced in recent years. Several large sample studies have indicated LG achieved similar or even shorter operation time compared with OG when surgeons have passed the learning curve [37–39], suggesting it may be a drawback of LG no longer in the future.

As a general benefit of laparoscopic techniques, the LG group was favored with less intraoperative blood loss. High heterogeneity between groups was observed in our study, which was likely to relate with the diversity of surgeons’ experience and methods to estimate the blood loss. This notable benefit mainly attributed to the nature of laparoscopic techniques. Magnified operation view facilitates meticulous manipulation. On the other hand, using harmonic instruments contribute to dissecting vessels around the stomach precisely and efficiently [40]. Less blood loss during LG may help maintain cardiopulmonary function stability and reduced the subsequently potential risk of postoperative morbidity.

Because none of the pooled studies reported the hazard ratios and its 95 % CI and the Kaplan-Meier curves in several pooled studies were of poor quality, we did not analyze the pooled 5-year OS rate. However, all three pooled studies reported that a 5-year OS rate of LG was comparable with OG. Though indirectly, the number of retrieved lymph nodes is usually used as an indicator of the oncologic adequacy of gastrectomy. In our study, no significant difference of retrieved lymph nodes was observed between two groups. The number of retrieved lymph nodes in both the LG and OG group was more than 15 as recommended [41], which was considered to be oncologically acceptable. The extent of lymphadenectomy remains controversial, though D2 lymphadenectomy has been reported to yield better prognostic outcomes [42–44]. In elderly patients, surgeons are usually reluctant to perform D2 resection to avoid major postoperative complications. Takeshita et al. reported that radical lymph node dissection for elderly patients may reduce life expectancy, especially in stage I and II patients [27]. They also recommend that R0 resection with at least limited lymph node dissection according to the Japanese guideline should be considered as the first choice of treatment for this population. It was actually reported that there are no significant benefits of D2 over D1 for patients >70 years old (5-year OS 19.8 % for D2 and 23.1 % for D1; *P* > 0.05) [45]. The average life expectancy of elderly patients is short, which may obscure the value of D2 lymphadenectomy. Therefore, more well-designed studies need to evaluate the proper extent of lymph node dissection in elderly patients.

Our studies also had some limitations, which should be taken into consideration before clinical practices. First, there was no randomized controlled study included in this study. Potential bias may exist in the selection of patients into the LG and OG group. Second, heterogeneity in studies with different cutoff age of elderly patients may also decrease the plausibility of the results. Third, the overall sample size of our study remained limited, and the inclusion of some small sample size studies or the method to estimate means and SDs described by Hozo may also result in bias. Fourth, this meta-analysis only included studies published in English or Chinese which may omit some important studies in other languages. Other biases may lie in that all pooled studies were from East Asia while no article comparing the LG and OG from other regions was retrieved. Nevertheless, Singh et al. reported that Western elderly patients could also undergo laparoscopic gastrectomy with low postoperative morbidity rate (3/20), suggesting the superior safety of LG in elderly patient [46].

## Conclusions

In conclusion, LG is a feasible and safe approach for elderly patients with gastric cancer. Compared with its open counterpart, LG has less blood loss, faster postoperative recovery, and reduced postoperative morbidity.
